# Synergistic Anticancer Activity of Fucoidan from *Lessonia trabeculata* Combined with Chemotherapeutic Agents in 4T1 Breast Spheroids

**DOI:** 10.3390/md23120451

**Published:** 2025-11-26

**Authors:** Rosa María Condori Macuri, Libertad Alzamora-Gonzales, Erasmo Honorio Colona-Vallejos, Raisa Teresa Cruz Riquelme, Laura Inés Pecho Chávez, Jherson Oscar Cisneros Gutierrez, Victor Alonso Montejo Anlas

**Affiliations:** Research Group Immunomodulators and Antitumor of Natural and Synthetic Origen, Immunology Laboratory, Universidad Nacional Mayor de San Marcos, Lima 11-0058, Peru; ecolonav@unmsm.edu.pe (E.H.C.-V.); raisa.cruz@unmsm.edu.pe (R.T.C.R.); laura.pecho@unmsm.edu.pe (L.I.P.C.); jherson.cisneros@unmsm.edu.pe (J.O.C.G.); victor.montejo1@unmsm.edu.pe (V.A.M.A.)

**Keywords:** fucoidan, *Lessonia trabeculata*, triple-negative breast cancer, Chou–Talalay method, synergistic effect, oxidative stress, 4T1 spheroids

## Abstract

Triple-negative breast cancer (TNBC) is known for being aggressive and potentially resistant to chemotherapy. This means that new ways to improve cancer treatments are a priority. So, the anticancer effect of binary combinations of fucoidan (FuLt) and the chemotherapeutic agents doxorubicin, paclitaxel, and 5-fluorouracil was evaluated. The Chou-Talalay combination index method was used to do this. This method allows the assessment of interactions between products by determining synergism, additive effect and antagonism with combination index <1, =1 and >1, respectively. Synergistic indices (SIs) were selected and applied to 4T1 homotypic spheroids. Oxidative stress caused by SIs was determined after 72 h by measuring the production of ROS and NO in both homotypic and heterotypic spheroids. Three SIs with an inhibitory effect of at least ≥ 0.50 and a dose reduction index > 1 were selected. Considering the experimental and simulated SI, twelve, nineteen, and seven SIs were found for FuLt with doxorubicin, paclitaxel, and 5-fluorouracil, respectively. The highest levels of ROS and NO occurred at 12 and 72 h, respectively, in homotypic and heterotypic spheroids, indicating an immunomodulatory effect in heterotypic spheroids. These results suggest that the synergistic combination of FuLt with chemotherapeutic agents improves drug efficacy and modulates redox dynamics in 4T1 spheroids. Furthermore, FuLt alone exhibits cytotoxic properties.

## 1. Introduction

Breast cancer is the most common malignant neoplasm in the female population worldwide, with around 2.3 million new cases reported each year [1]. Its incidence is expected to continue rising, with an estimated 29.5 million associated deaths predicted by 2040 [2]. Triple-negative breast cancer (TNBC) is a heterogeneous group of tumours with an aggressive clinical profile characterised by metastasis and recurrence. TNBC has the worst prognosis of all subtypes [3]. The absence of classical breast tumour markers (oestrogen receptors, progesterone receptors and human epidermal growth factor receptor 2) limits therapeutic options. Systemic chemotherapy using anthracyclines and taxanes is the most common treatment and cornerstone of clinical management [3,4]. In addition to these limited therapeutic options, patients face challenges such as drug resistance [4] and an impaired quality of life due to the adverse effects of treatment.

Some of the chemotherapy drugs commonly used for TNBC include doxorubicin (DOX), paclitaxel (PTX) and 5-fluorouracil (5-FU). DOX is an anthracycline which damages genetic material and alters iron and calcium metabolism, thereby inducing apoptosis and autophagy [5]. PTX, is a taxane derived from the yew tree. It causes cell cycle arrest by inhibiting mitotic spindle function, leading to apoptosis [6]. PTX is also characterised by its ability to induce autophagy in tumour stem cells, a process that has been identified as one of the causes of chemoresistance [6]. Similarly, 5-FU acts on actively dividing cells by inhibiting RNA replication and thymidylate synthase, ultimately blocking DNA replication [7]. However, this can lead to chemoresistance, as well as neurological and cardiac toxicity, myelosuppression, and other side effects.

Due to the limited treatment options available and the aggressive nature of the disease, developing effective therapies is crucial. These include combination therapeutic regimens, where the proper application of polychemotherapy can enhance treatment efficiency [8]. Adjuvant natural therapies have also been proposed to improve the effectiveness of chemotherapy, either by increasing its anticancer potential or by immunomodulation, which could reduce the medication’s harmful side effects [9].

Currently, a total of twenty-eight bioactive compounds with proven antitumor activity are undergoing clinical trials in phases I to III [10,11]. Fucoidan, a polysaccharide extracted from brown seaweed, stands out among the marine-derived compounds with scientific evidence supporting their anticancer and immunomodulatory properties [12]. Its composition varies and includes fucose, sulphate, uronic acid, galactose, xylose, mannose, rhamnose, glucose and arabinose. It has been shown to be non-toxic and is currently marketed as a functional supplement in several countries [13]. Fucoidan from *Lessonia trabeculata* (FuLt) has demonstrated cytotoxic activity against TNBC 4T1 cells in both monolayer and three-dimensional cultures [14], whilst reducing cytotoxicity against VERO cells [15,16]. Furthermore, FuLt has been shown to be non-toxic to both RAW264.7 cells [17] and human peripheral blood mononuclear cells [18].

Numerous studies have explored the use of fucoidan in chemotherapics combinations against various types of neoplastic cells, reporting enhanced cytotoxic activity, increased apoptosis, reduced tumour cell migration and decreased recurrence in both preclinical [19,20,21,22,23] and clinical trials [24]. The synergistic effect of two or more compounds is more than just the sum of their individual effects. Rather, it requires a more specific analysis based on their kinetics and dynamics [25]. Therefore, we consider it necessary to explore and validate the possible synergy between FuLt and conventional chemotherapeutic drugs through mathematical analysis that supports their combined use.

Reactive oxygen species (ROS) and reactive nitrogen species (RNS) play an important role in the tumour microenvironment [26]. Excessive intracellular ROS accumulation can have anticancer effects, including the inhibition of the cell cycle, the suppression of nucleotide and ATP synthesis, and the activation of pro-apoptotic pathways mediated by proteins such as p53 and caspases 9 and 3/7. It can also activate endoplasmic reticulum stress and induce ferroptosis [27]. Conversely, low concentrations of NO can promote angiogenesis and evasion of the immune response, whereas high levels can promote apoptosis, cellular senescence, and immunogenic cell death [28].

Following on from our previous findings of cytotoxicity and immunomodulation on a TNBC 4T1 3D model using fucoidan from *L. trabeculata* and a DOX [14]. This study aimed to determine the synergistic activity of the combination of FuLt and three chemotherapeutic drugs (DOX, PTX and 5-FU) in homotypic TNBC 4T1 spheroids. We also investigated the oxidative effect of these combinations on both homotypic and heterotypic spheroids (4T1 cells and mouse splenocytes). Both the experimental and simulated doses were obtained using the Chou-Talalay method. FuLt + PTX was found to be the most effective combination, providing the highest selectivity index (SI) at the lowest dose. Furthermore, FuLt’s pro-oxidant capacity was revealed through increased ROS and NO levels in both homotypic and heterotypic spheroid cultures. These results emphasise the importance of considering redox balance as a critical factor in the efficacy of combined anticancer therapies, as well as its potential link with cytotoxicity. It is recommended that the SI approach be applied to an in vivo TNBC 4T1 model in order to evaluate its potential progression to clinical trials.

## 2. Results

### 2.1. Effect of FuLt on the Viability of Homotypic TNBC 4T1 Spheroids

**Figure 1:**
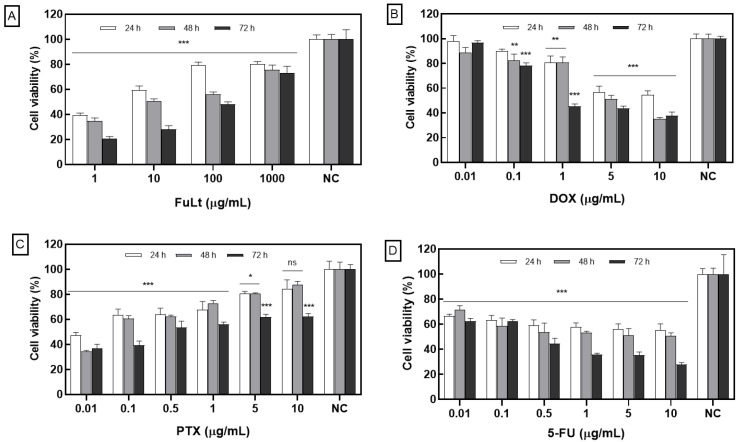
Cytotoxic effect of fucoidan and chemotherapeutic agents against 4T1 triple negative breast cancer (TNBC) spheroids: (**A**) fucoidan from *L. trabeculata* (FuLt), (**B**) doxorubicin (DOX), (**C**) paclitaxel (PTX) and (**D**) 5-fluorouracil (5-FU) for 24 h, 48 h and 72 h. Data are presented as mean ± SD (All experiments were performed in quadruplicate). *** *p* < 0.001; ** *p* < 0.01; * *p* < 0.05; ns = not significant; NC: negative control (untreated spheroids).

A strong cytotoxic effect was obtained for FuLt-treated 4T1 spheroids at concentrations of 1, 10, 100 and 1000 μg/mL compared to NC (* *p* < 0.05, ** *p* < 0.01, *** *p* < 0.001) in evaluations performed after 24, 48 and 72 h (Figure 1A).

Spheroids treated with 1, 5 and 10 μg/mL of DOX exhibited significant, concentration-dependent cytotoxicity at the evaluated time points. In contrast, 0.1 μg/mL only proved effective after 48 h of treatment (Figure 1B).

PTX inhibited spheroid viability at all three evaluated time points. In contrast, a concentration of 10 μg/mL only reduced viability after 72 h of treatment (Figure 1C). 5-FU produced significant cytotoxicity compared to the NC (* *p* < 0.05, ** *p* < 0.01, *** *p* < 0.001) and exhibited a concentration-dependent effect (0.01–10 μg/mL) at all three time points (Figure 1D). Cytotoxicity for FuLt and PTX showed an inverse relationship to concentration.

### 2.2. Half-Maximal Inhibitory Concentration (IC_50_)

**Figure 2:**
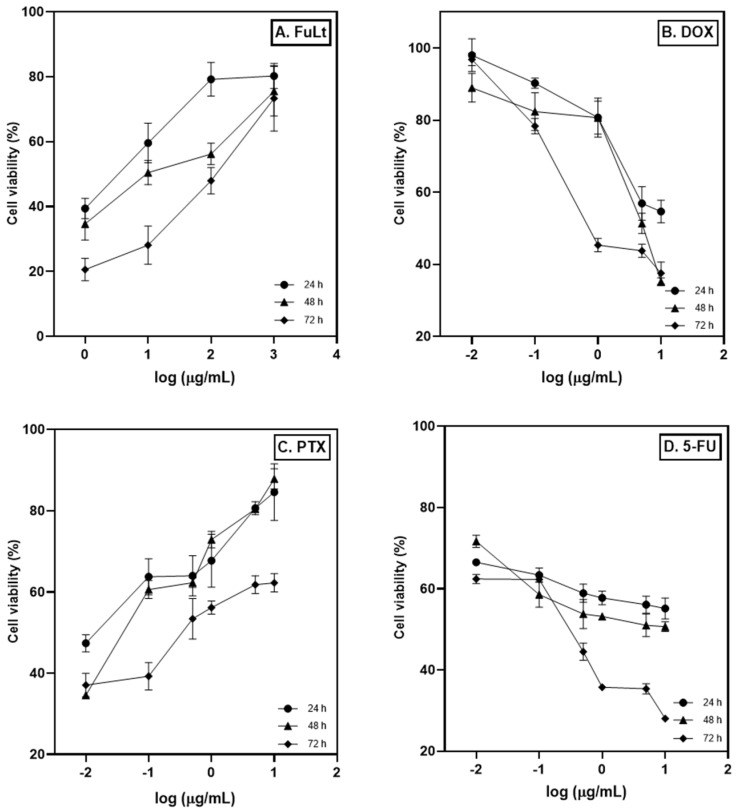
Half-maximal inhibitory concentration (IC_50_) of TNBC 4T1 spheroids. (**A**) fucoidan from *L. trabeculata* (FuLt), (**B**) doxorubicin (DOX), (**C**) paclitaxel (PTX) and (**D**) 5-fluorouracil (5-FU). Data are presented as the mean ± SD (All experiments were performed in quadruplicate).

The IC_50_ values for FuLt, DOX, PTX and 5-FU on 4T1 spheroids were determined at 24, 48 and 72 h using GraphPad Prism software (Table 1). The IC_50_ values were obtained based on the logarithm concentration of the treatments (µg/mL) versus the spheroids’ percentage cell viability (Figure 2). These values indicate the performance and cytotoxic potency of FuLt and the chemotherapeutic agents used. Potency is defined as the concentration of a compound required to produce a 50% reduction in cell viability. Therefore, the results from Table 1 can be expressed as PTX > 5-FU > DOX > FuLt.

### 2.3. Cytotoxic Effect Based on the Chou-Talalay Method

Cytotoxicity data were obtained by performing an MTT assay on each drug against TNBC 4T1 spheroids. Concentration-response curves were then generated using CompuSyn software version 1.0, and the following parameters were calculated: the drug-affected fraction (*fa*), the drug potency (*Dm*), the shape of the concentration-response curve (*m*) and the linear correlation coefficient (r), as described in the original equation by Chou and Talalay [25] (Table 2). Extended data are shown in the Supplementary Materials.

As shown in Table 2, all treatments inhibited cell growth. The *Dm* values (corresponding to the IC_50_) of FuLt, DOX, PTX and 5-FU for all time points evaluated are almost identical to those obtained using GraphPad Prism software.

### 2.4. SI for FuLt and Three Chemotherapeutics Against TNBC 4T1 Spheroids

**Figure 3:**
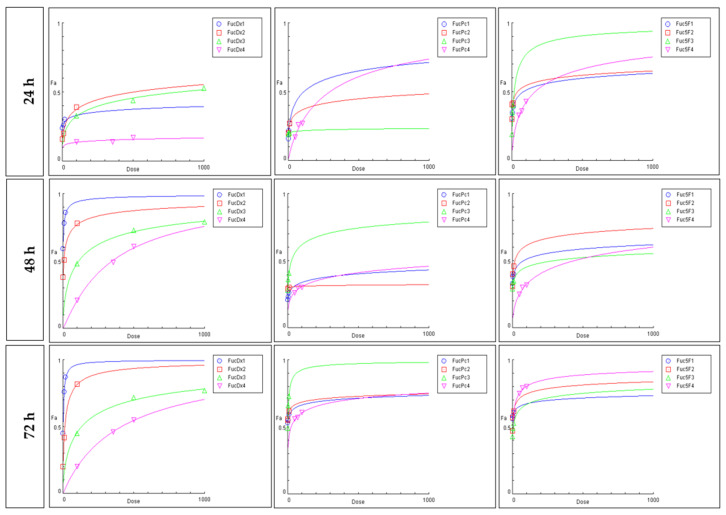
Concentration-effect curves for binary combinations between FuLt and three chemotherapeutic agents. FucDx = ratio between fucoidan from *L. trabeculata* and doxorubicin, FucPc = ratio between fucoidan from *L. trabeculata* and paclitaxel, Fuc5F = ratio between fucoidan from *L. trabeculata* and 5-fluorouracil. Where *m* > 1 is sigmoidal, *m* < 1 is flat sigmoidal and *m* = 1 is hyperbolic. *fa*: fraction inhibition, *m*: slope of the median effect plot.

MTT cytotoxicity assays were performed for different binary combinations at three time points to provide an in vitro analysis of the pharmacological interaction (synergism, antagonism or additivity interactions) between FuLt and the selected antineoplastic agents. Figure 3 shows the concentration-response curves generated for each binary combination. In all cases, the curve was flat sigmoidal (*m* < 1), except for the 175:1 ratio for FuLt + DOX at 48 h of treatment. All r values of the curves were greater than 0.94, indicating compliance with the law of mass action, with r = 1 representing complete or ideal compliance [25].

Combination ratios were proposed based on the *fa* values of each treatment (Table 2). Three combination scenarios were considered for each chemotherapy drug and FuLt. Four experimental combination concentrations were selected and evaluated after 24, 48 and 72 h. The results are presented in Table 3, Table 4 and Table 5, and the raw data for each binary combination can be found in the Supplementary Materials.

Combinatorial concentrations with a synergistic effect (CI < 1) are numbered from one to twenty-five (superscript) in Table 3, Table 4 and Table 5. Six SIs were identified for FuLt + DOX, fourteen for FuLt + PTX, and five for FuLt + 5-FU.

Only SI^3^ and SI^20^ exhibited a synergistic effect at *fa* ≥ 0.5, suggesting a pharmacodynamic interaction that can inhibit 50% of spheroid-forming cells. Additionally, they have a DRI > 1 for at least one of their components. This suggests that, when combined, the concentrations of chemotherapeutic agents used with fucoidan are lower than those used in non-combination treatments. This makes SI^3^ and SI^20^ the most promising experimental SI.

At the same time, the CompuSyn software version 1.0 generates simulated combinatorial doses from the processed experimental data (Tables S1–S3). These tables summarise the simulated pharmacodynamic interactions with *fa* ≥ 0.5. Six simulated SI values were obtained for FuLt + DOX, five for FuLt + PTX, and two for FuLt + 5-FU. The sum of the experimental and simulated doses yielded twelve for FuLt + DOX, nineteen for FuLt + PTX, and seven for FuLt + 5-FU. FuLt + PTX is the most promising combination, presenting the highest number of SIs with lower total doses.

**Figure 4:**
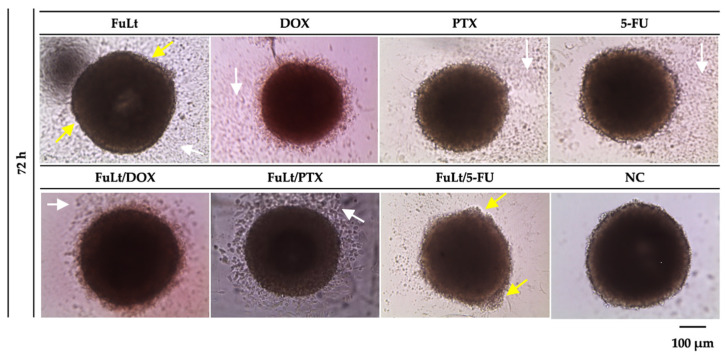
Cytotoxic effects of *L. trabeculata* fucoidan (FuLt), doxorubicin (DOX), paclitaxel (PTX) and 5-fluorouracil (5-FU), as well as three syner-gistic combinations, were examined on 4T1 spheroids after 72 h of treat-ment. The negative control (NC) refers to untreated spheroids with an av-erage diameter of 440 µm ± 0.02. The white arrows indicate significant cellular debris around the spheroids. The yellow arrows indicate defor-mation of the spheroids.

We observed the cytotoxic effects of the treatments applied using light microscopy. These effects included cell detachment from the spheroids and deformation of the spheroids themselves (Figure 4).

### 2.5. Oxidative Stress Caused by FuLt, Three Chemotherapeutic Agents and Three SIs in Homotypic and Heterotypic Spheroids

**Figure 5:**
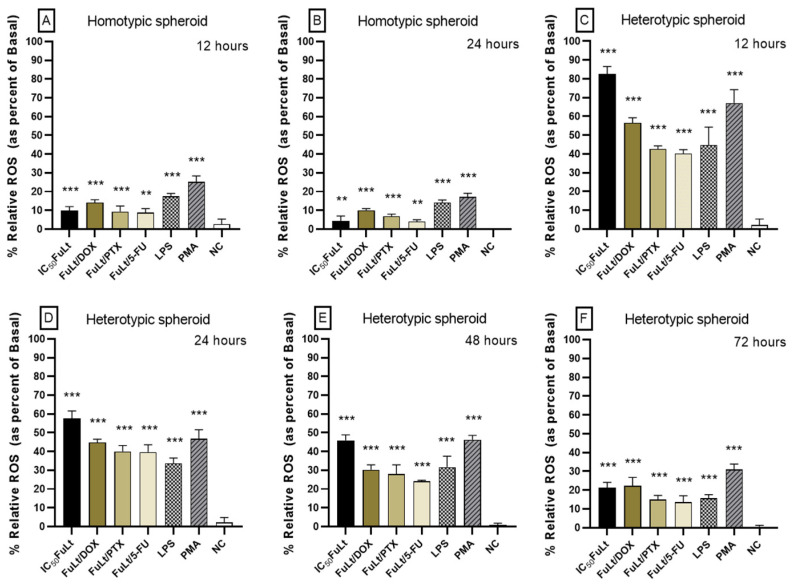
Relative ROS production in 4T1 homotypic and heterotypic spheroids (splenocytes + 4T1 cells), treated with IC_50_ dose of FuLt and with DOX, PTX and 5-FU synergistic doses. (**A**,**B**) in 4T1 homotypic spheroids; (**C**–**F**) in heterotypic spheroids. FuLt: Fucoidan from *L. trabeculate*); DOX: doxorubicin; PTX: paclitaxel; 5-FU: 5-fluorouracil. Positive controls were used as oxidative stress inducers: LPS (*Escherichia coli* lipopolysaccharide) and PMA (phorbol myristate acetate). NC: negative control (untreated spheroids). Data are presented as mean ± SD (*n* = 6, the number of mouse spleens used is represented by *n*). *** *p* < 0.001; ** *p* < 0.01.

The relative production of ROS in homotypic spheroids (Figure 5A,B) treated with SI was higher after 12 h than after 24 h (*p* < 0.001 and *p* < 0.01, respectively). From 48 h onwards, low and insignificant amounts of ROS were produced compared to baseline production and were not plotted. In heterotypic spheroids (Figure 5C–F), ROS production decreased gradually from 12 to 72 h of treatment (*p* < 0.001). ROS production was higher in heterotypic spheroids than in homotypic spheroids.

**Figure 6:**
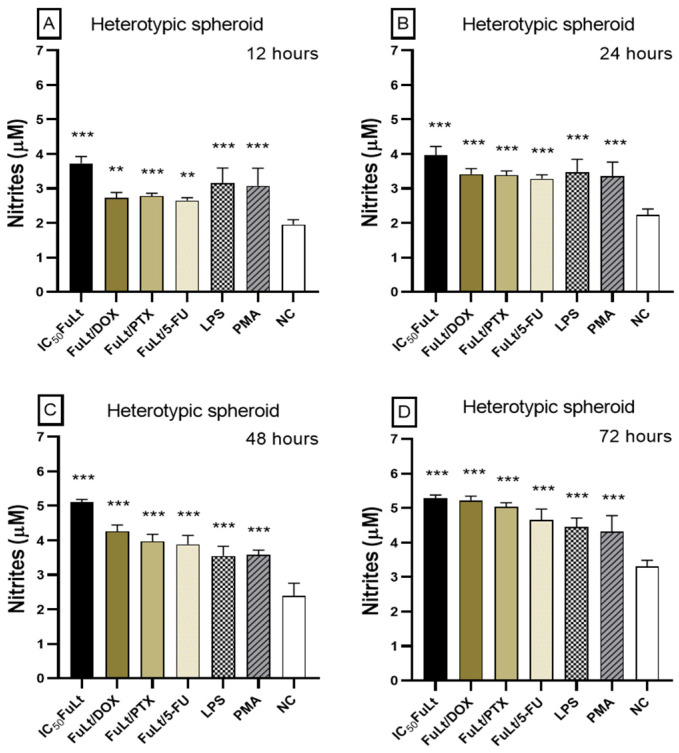
Nitrites production in heterotypic spheroids of splenocytes and 4T1 cells treated with IC_50_ FuLt: Fucoidan from *L. trabeculata* and synergistic doses between FuLt and DOX: doxorubicin, PTX: paclitaxel and 5-FU: 5-fluorouracil. (**A**) 12 h, (**B**) 24 h, (**C**) 48 h and (**D**) 72 h. LPS: lipopolysaccharide from *Escherichia coli* and PMA: phorbol myristate acetate were used as inducers of oxidative stress. NC: negative control (untreated spheroids). Data are presented as mean ± SD (*n* = 6). *** *p* < 0.001; ** *p* < 0.01.

Furthermore, non-significant increase in nitric oxide (NO) was observed in homotypic spheroids treated with the SIs of the three chemotherapeutic agents. In heterotypic spheroids, the concentration of NO increased after 12 h, reaching its maximum after 72 h (Figure 6A–D) (*p* < 0.001 and *p* < 0.01). Additional assays with homotypic spheroids revealed an increase in NO with FuLt IC_50_ (*p* < 0.001) at 12, 24, 48 and 72 h (Figure S2).

## 3. Discussion

Accumulating evidence on the anticancer effects of fucoidans extracted from brown algae has prompted further study of FuLt [14,15,16]. This approach addresses one of the main challenges in treating this type of cancer: chemoresistance [29].

The cytotoxic potential of FuLt was evaluated individually in homotypic TNBC 4T1 spheroids. The observed cytotoxicity at concentrations between 1 and 1000 µg/mL is consistent with previous studies on fucoidans from other brown algal species against breast cancer cell. For example, studies on human breast cancer T-47D cells have reported similar results with *Alaria* sp., *Saccharina japonica* [30,31], and *Undaria pinnatifida* [31] at concentrations between 200 and 800 µg/mL. Fucoidan extracted from *Undaria pinnatifida* has been demonstrated to exhibit cytotoxic properties at 200 to 1000 µg/mL against MCF-7 cells [32]. Similarly, fucoidan derived from *Fucus vesiculosus* has been shown to exhibit cytotoxic properties against MDA-MB-231 cells at 90 to 120 µg/mL [33], 50 to 100 µg/mL [34], and 100 to 1600 µg/mL [22]. Fucoidan from *F. vesiculosus* (90 to 200 µg/mL) was cytotoxic against 4T1 cells [33,35,36]. And Fucoidan from *Laminaria japonica* (400 µg/mL) was cytotoxic against MDA-MB-231 cells [37]. All these studies were conducted in monolayer cultures. Previous studies by our research group revealed the cytotoxic effects of FuLt on 4T1 cell monolayers at concentrations of 1000–10,000 µg/mL [15,16], while concentrations of 1–10,000 µg/mL were effective in 4T1 spheroids [14]. Chen et al. reported Fucoidan from *Laminaria japonica* treatment prevented MDA-MB-231 mammosphere formation in a dose-dependent manner (500 to 1000 µg/mL) [37].

Chemical properties could be the reason for the differences in cytotoxic potency between fucoidans from different seaweed sources. The presence of sugars, the concentration and position of sulphated residues, and the molecular weight could all directly influence biological activity [31]. FuLt presents 59% of total sugars and 5.7% of sulphates. Mak et al. extracted fucoidan from *Undaria pinnatifida* (New Zealand) with a significantly higher sulphate content (15%) and a uronic acid content of 1.24%, but did not specify the total sugar content [32]. Hsu et al. [33], Chen et al. [34], Xue et al. [35,36] and Zhang et al. [22] all used a commercially available fucoidan from *F. vesiculosus* with a sulphation level of 7–11%. No data on total sugars were provided. Meanwhile, Chen et al. used fucoidan with a higher sulphate content (35.4%) [37]. Vishchuk et al. [31] observed a close relationship between the position and quantity of sulphate groups and the greater cytotoxic effect of fucoidan from *U. pinnatifida* (54% total sugars, 29% sulphates) relative to that from *S. japonica* (56% total sugars, 23% sulphates). The balance between moderate sulphate levels and a high total sugar content may be key to FuLt’s robust antitumor activity in the breast cancer model used, even though its sulphation level is lower compared to other extracts. However, further structural characterisation (e.g., of the specific sulphation pattern, branching, and monosaccharide distribution) is crucial to better understand the impact of these variables on its mechanism of action.

FuLt exhibited an inverse relationship between its concentrations and its cytotoxic activity. Lower concentrations were more cytotoxic than higher ones. This relationship was previously reported by Condori et al. in TNBC 4T1 spheroids [14]. One possible explanation is the hormesis effect, characterised by increased efficacy at low concentrations and reduced efficacy at higher doses [38]. This effect has been documented for analogues such as fucoxanthin in fibroblast monolayers [39], for compounds such as resveratrol in human umbilical vein endothelial monolayers [40], and for curcumin in both human bladder [41] and normal fibroblast monolayers [42].

A study investigating the effects of *Fucus evanescens* fucoidan on malignant melanoma SK-MEL-28 cells revealed significant differences in treatment outcomes between two- and three-dimensional cell cultures. The study revealed that fucoidan prevented proliferation of SK-MEL-28 cells in monolayer but had no effect on three-dimensional cell cultures [43]. The present study provides data on the anticancer efficacy of FuLt against TNBC 4T1 cells, which is comparable to that reported by other researchers [30,31,32,33,34,35,36,37]. The advantage of FuLt is that it is also effective in a three-dimensional 4T1 model.

Nováková et al. demonstrated the synergistic effect of fucoidan and glycosaminoglycans on the cytotoxicity of homotypic Hepa-1c1c7 hepatocellular carcinoma spheroids [44]. The literature does not describe any synergistic effects between FuLt and the three chemotherapeutic agents tested on 4T1 cells or other TNBC cell lines, but there are reports indicating increased toxicity when fucoidans from other brown algal species are combined with DOX on breast cancer cells such as MDA-MB-231 [21,22], 4T1 [21] and MCF-7 [22] in monolayer. However, antagonistic interactions between *Fucus vesiculosus* fucoidan and DOX have also been reported in breast, ovarian and cervical cancer cells [20]. These interactions were found using Tallarida’s method.

FuLt and PTX treatments produced the highest number of SI. Our finding is consistent with the observed synergy between fucoidan from *Undaria pinnatifida* and PTX, and fucoidan from *Fucus vesiculosus* and PTX, in ovarian, cervical, and different breast cancer cell lines in monolayer, as determined using Tallarida’s method [20]. Using the Jin method of the modified probability sum test [45], Obluchinskaya et al. also reported synergy between fucoidan from *Fucus vesiculosus* and PTX in HeLa G-63 cells [23]. Moreover, it has been demonstrated that different concentrations of fucoidan from *Cladosiphon navae-caledoniae* in combination with PTX increase the level of cytotoxicity in MDA-MB-231 and MCF-7 [46]. However, synergy was not calculated.

The study by Huang et al. involving human colorectal cancer cells revealed that the anticancer activity of *Sargassum hemiphyllum* fucoidan improved when combined with 5-FU [19]. Furthermore, a clinical trial involving patients with unresectable advanced colorectal cancer demonstrated that those who received *Cladosiphon okamuranus* fucoidan in combination with 5-FU and oxaliplatin experienced fewer chemotherapy-related adverse effects [24]. In both studies, the synergy of the treatments was not calculated. Our findings demonstrate that FuLt can interact synergistically with conventional chemotherapeutic drugs DOX, PTX and 5-FU. Identifying synergistic combinations using a quantitative approach strengthens the scientific basis for considering FuLt as an adjuvant in combination therapies, helping to overcome chemoresistance.

Three SIs were validated by quantifying ROS and NO in homotypic 4T1 spheroids and heterotypic (4T1 + splenocytes) spheroids. In homotypic spheroids, ROS production at 12 and 24 h would indicate the early activation of oxidative stress following treatment. The IS were validated by quantifying ROS and NO in both homotypic and heterotypic (4T1 + splenocytes) spheroids. ROS production was observed at 12 and 24 h in homotypic spheroids, indicating early activation of oxidative stress following exposure to the treatments. Studies using cell monolayers, such as that conducted by Banafa et al. on MCF-7 cells, have shown an increase in ROS production 24 h after fucoidan treatment from *Fucus vesiculosus* [47]. Similarly, Zhang et al. obtained a peak in ROS production at 30 min when using fucoidan from *Cladosiphon novae*-*caledoniae* on the same cell line, and demonstrated its relationship with the activation of MAPK and Bcl-2-dependent apoptotic pathways [48]. Fucoidan from *Fucus vesiculosus* has been reported to increase ROS in the following cell lines: ES-2 and OV-90 (ovarian cancer) [49]; CAL27 and Ca9-22 (oral carcinoma) [50]; and SKM-1 (leukaemia) [51].

Regarding combination treatment, Zhang et al. [46] used fucoidan from *Cladosiphon novae-caledoniae* and PTX, demonstrating an increase in ROS in MDA -MB-231 and MCF-7 monolayers after 48 h. However, the present study, which used both homotypic and heterotypic spheroids, observed ROS depletion after 48 h of treatment. This suggests either early oxidative activity and depletion at the time of quantification, or the activation of intracellular antioxidant systems by tumour cells [52].

In treatments involving three SIs on spheroids, heterotypic cells exhibited significantly higher ROS levels than homotypic cells after 12 and 24 h. This suggests that immune cells in the tumour microenvironment may be able to amplify the oxidative response. It has been demonstrated that activated splenocytes, which consist mainly of macrophages, neutrophils and T cells, increase the pro-oxidant component by producing hydrogen peroxide and superoxide [52], as well as cytokines such as IL-1β, IL-6 and TNF-α, which activate ROS-dependent cell death pathways [53]. Culturing human neutrophils with 10 µg/mL of fucoidan from *Undaria pinnatifida* increased the production of the pro-inflammatory cytokines IL-1β, IL-6, IL-8 and TNF-α after 24 h [54]. In murine macrophage cultures treated with 100 µg/mL of 98% pure commercial fucoidan, IL-1β production increased after 4 h [55]. Kar et al. found that ROS production increased in macrophages obtained ex vivo from mice infected with *Leishmania donovani* and subsequently treated with 50 µg/mL of fucoidan from *F. vesiculosus* for 48 h [56].

In addition to reactive oxygen species, other redox mediators, such as NO, which is an inducer of nitrosative stress, play a key role in the cellular response to stress. In the homotypic spheroid, evidence of a production profile from lower to higher NO concentration was only observed in the FuLt treatment between 12 and 72 h, which is related to the antitumor activity of fucoidan. Similarly, Jin et al. reported an increase in NO production after 48 h in human leukaemia cells treated with fucoidan (150 g/mL) from *F. vesiculosus* only [57]. In addition, they confirmed that fucoidan activates caspases 3, 8 and 9 in the presence of NO. This suggests that apoptosis is one of the mechanisms by which fucoidan induces cell death.

Similarly to our findings, the highest NO production occurred after 72 h of treatment with the three SI. Other authors have reported increased NO levels in mouse splenocytes treated with fucoidan from *Undaria pinnatifida* and *Fucus vesiculosus* after 48 h [58] and 96 h [59], respectively. The difference in our assays is that we used heterotypic spheroids, in which the immune cells present in the splenocytes that form part of the tumour immune microenvironment enhance the antitumor response [60]. In addition, these authors reported increased levels of IFN-γ, TNF-α and IL-6. The latter two of these cytokines are related to the induction of NO production [61].

Overall, the results obtained reveal a redox compensation phenomenon whereby sustained NO production indicates an immunomodulatory response that offsets the initial oxidative damage caused by ROS. This is due to the cytotoxicity exhibited by FuLt and the IC_50_ values of the three chemotherapeutic agents evaluated on homotypic and heterotypic spheroids.

Despite the promising results, the study has limitations. While it is important to determine the presence of certain secondary metabolites, such as polyphenols and terpenoids, the concentration of fucoidan used in the study due to the extraction method means that the antineoplastic effect can be attributed to this product. Although the FuLt used is ultrafiltrable, its molecular weight must be accurately determined. In vivo model trials should also evaluate the pharmacokinetics of FuLt, including parameters such as dissolution, bioavailability, distribution, metabolism and elimination.

## 4. Materials and Methods

### 4.1. Chemicals

According to data provided by Peruvian Seaweeds SA (PSW SA) (Lima, Peru), specimens of *L. trabeculata* were collected in San Nicolás Bay (15°15′39″ S, 75°13′47″ W), Marcona district, Nasca province, Peru. The fucoidan (FuLt) obtained from this seaweed was 83.4% pure and contained 59% of total sugars and 5.7% of sulphates. The cation content (g%g) was as follows: Na^+^ (0.71%), K^+^ (0.65%), Ca^2+^ (7.1%) and Mg^2+^ (0.33%). The fucoidan was obtained by acid extraction. The lyophilised fucoidan was dissolved in RPMI-1640 medium supplemented with 10% foetal bovine serum (cRPMI). It was then treated with ultrasound (Qsonica Q55 model) and sterilised by filtration using 0.22 µm membranes to produce ultrafiltrable fucoidan.

Reagents of analytical grade were purchased from Sigma-Aldrich (St. Louis, MO, USA): Doxorubicin hydrochloride (cat# D1515); paclitaxel (cat# T7191), 5-fluorouracil (cat# F6627), sodium bicarbonate (cat# S5761), penicillin–streptomycin solution (cat# P4333), Dimethyl sulfoxide (DMSO, cat# 472301), 3-(4,5-Dimethyl-2-thiazolyl)-2,5-diphenyl-2H-tetrazolium bromide (MTT reagent, cat# M5655), sodium pyruvate (cat# P5280), Nitro blue Tetrazolium Chloride (NBT, cat# N6876), lipopolysaccharides from *Escherichia coli* 0111:B4 (LPS, cat# L4130), and phorbol myristate acetate (PMA, cat# P1585).

Reagents purchased from GIBCO (New York, NY, USA) were RPMI-1640 (cat# 31800-022) and ACK lysis buffer (cat#A10492-01). Fetal Calf Serum (FCS) was purchased from Biowest (Bradenton, FL, USA, cat# S1620), Trypsin-EDTA was purchased from Pan Biotech (Aidenbach, Germany, cat# P10-023500), Amphotericin B was purchased from Gibco (Paris, France, cat# 15290018), Griess Reagent System was purchased from Promega (Fitchburg, WI, USA, cat# G2930), and Ketamine/xylacine Injectable Solution for veterinary use was purchased from Ket-A-Xyl^®^ (Lima, Peru).

### 4.2. Animals

Six nulliparous female BALB/c mice, aged six weeks and weighing approximately 20 ± 2 g, were purchased from the Instituto Nacional de Salud (Lima, Peru) for the in vitro experiments. The mice were acclimatised for five days in the Biotherium of the Faculty of Biological Sciences (Universidad Nacional Mayor de San Marcos, Lima, Perú). They were fed water and food (commercial dry pellets for mice) *ad libitum*, and kept under consistent temperature conditions (23 ± 1 °C) and a 12:12 light/dark photoperiod.

The animals were anaesthetised with a lethal dose of 300 µL ketamine/xylacine (100 mg/20 mg/kg body weight). Their spleens were removed to isolate splenocytes for the formation of heterotypic spheroids. All experimental procedures adhered to the regulations for using laboratory animals, as outlined by the Bioethics Committee of Faculty of Biological Sciences (Code:059-2023-CBE-FCB-UNMSM, Universidad Nacional Mayor de San Marcos, Lima, Peru).

### 4.3. Cell Culture Conditions

The 4T1 cell line is an epithelial lineage which adheres to surfaces and is known to develop the characteristic triple-negative mammary tumour phenotype in mice. This cell line was obtained from the Cell Bank of Rio de Janeiro, Brazil (code BCRJ0022). The cells were cultivated in cRPMI consisting of 0.2% sodium bicarbonate, 1% sodium pyruvate, 1% penicillin–streptomycin and 0.05% amphotericin B, in accordance with standard culture conditions (37 °C, 95% humidity and 5% CO_2_).

### 4.4. Formation of 4T1 Homotypic Spheroids

The cells were cultured in cRPMI in round-bottom microplates (1 × 10^4^ cells per well), which were coated with 50 µL of 1.5% agarose for 15 min, using the liquid overlayer method [31]. The plates were then centrifuged at 2000 rpm for 5 min to facilitate cohesion and spheroid formation. The plates were then incubated for 96 h under standard conditions without replacing the medium.

### 4.5. Formation of the Heterotypic Spheroid

For this assay, splenocytes were extracted from mice according to the protocol described by Nilofar Danishmalik et al. [62]. The spleen was cut into small pieces, after which the resulting suspension was passed through a 70 µm cell strainer. Red blood cells were removed from the cell suspension using lysis buffer, after which the suspension was centrifuged at 1500 rpm with cRPMI to remove debris. The same conditions were used for culturing 1 × 10^4^ cells per well in a final volume of 200 µL as for homotypic spheroids. The microplates were then centrifuged at 2000 rpm for 5 min and incubated under the same conditions as the homotypic spheroids [14]. The entire procedure was performed under sterile conditions.

### 4.6. Viability Assay

The MTT reduction assay was used to analyse spheroids treated with FuLt and DOX, PTX or 5-FU. The FuLt dilutions used were 1, 10, 100 and 1000 μg/mL. For the chemotherapeutic agents, the dilutions were 0.01, 0.1, 0.5, 1, 5 and 10 μg/mL. The spheroids were incubated with FuLt, DOX, PTX or 5-FU at the specified concentrations for 24, 48 or 72 h under standard conditions. The results represent the mean of four spheroids per condition in three independent experiments.

Untreated spheroids were used as the negative control. 50 μg/mL of MTT was added to each well. The wells were incubated for a further 4 h, after which the formazan crystals were diluted with 100 μL of DMSO per well. All experiments were performed in quadruplicate. Absorbance was measured at 570 nm (with a 630 nm reference) using a BioTek Epoch 2 spectrophotometer (Winooski, VT, USA).

### 4.7. Determination of the Half-Maximal Inhibitory Concentration (IC_50_)

Cell viability was calculated as a percentage using the following formula:

$$Cell\;viability\;\left(\%\;of\;control\right)=\left(100\times \frac{Absorbance\;of\;treated\;spheroids}{Absorbance\;of\;control\;spheroids}\right)$$

Concentration-effect inhibition curves were constructed from these values by plotting the logarithm of the concentrations against the percentage cell viability. The IC_50_ was obtained using GraphPad Prism software (version 8.01, San Diego, CA, USA) and the non-linear regression model.

### 4.8. Determining the Optimal Binary Combination Using the Chou-Talalay Method

The combination index (CI) indicates the type of drug–drug interaction that is occurring: CI = 1 represents an additive effect, CI < 1 represents a synergistic effect, and CI > 1 represents an antagonistic effect [19].

To determine whether the proposed treatments exhibit synergism, antagonism or additivity, the cytotoxicity data obtained in Section 4.4 were calculated individually using the following formula:

$$Cytotoxicity=\left(1-\frac{Absorbance\;of\;treated\;spheroids-Absorbance\;of\;blank}{Absorbance\;of\;control\;spheroids-\;Absorbance\;of\;blank}\right)$$

These data were entered into CompuSyn software version 1.0 (ComboSyn, Inc., Paramus, NJ, USA) to generate dose-effect curves showing the pharmacodynamic interactions of the two compounds, based on the median effect equation from the Chou–Talalay method.

$$\frac{fa}{fu}=\left({\frac{D}{Dm}}^{m}\right)$$

*fa* corresponds to the fraction of the cells affected by the drug, and fu corresponds to the fraction of the cells unaffected by the drug (*fu* = 1 − *fa*). The ratio of the two is equal to the drug concentration or dose (*D*). Drug potency is represented by ‘*Dm*’, where ‘*m*’ represents the shape of the dose-effect curve (i.e., sigmoidal or hyperbolic) [20]. The degree of conformity of the data to the law of mass action is indicated by the r-value. If r = 1, this is known as ideal conformity [19].

The unified equation, the mean effect equation of the mass action law developed by Chou and Talalay, allows the derivation of more than 300 enzyme dynamics equations to be summarised [19], thus giving a mathematical basis for the effects shown by drugs used in combination.

Only the effective combinatorial doses produced by CompuSyn software version 1.0 that resulted in 50% cytotoxicity of the spheroids and were synergistic were chosen.

### 4.9. Oxidative Activity

To evaluate oxidative stress, one SI was selected for each combination of FuLt with a chemotherapeutic agent. SIs obtained at 72 h with *fa* ≥ 0.5 and DRI > 1 in at least one component were chosen. The SIs tested were: FuLt-DOX (ratio 1:1, total dose 2 µg/mL), FuLt-PTX (ratio 1:1, total dose 1 µL) and FuLt-5-FU (ratio 2:1, total dose 0.33 µL). Phosphatidylinositol myristoyl myo-inositol 3-phosphate 4-phosphatidylinositol (PMA) and *Escherichia coli* lipopolysaccharide (LPS) were used as oxidative stress inducers. The assays were performed using homotypic and heterotypic spheroids (one spheroid per well) for 72 h (*n* = 6).

ROS quantification was performed by NBT reduction, according to the method described by Blockhuys et al. The spheroids were transferred to a flat-bottomed microplate and washed twice with 150 µL of 1× PBS to remove any residual cRPMI. Then, 30 µL of NBT was added to each well and the plate was incubated at 37 °C for 120 min, protected from light. The plate was then washed with PBS and the formazan crystals dissolved with 120 µL of 2 M KOH and 140 µL of DMSO. Absorbance was measured at 630 nm [63]. The percentage of NBT reduction was calculated using the following formula:

$$\%\;NBT\;cell\;reduction=\frac{OD\;sample-OD\;negative\;control}{OD\;negative\;control}\times \;100$$

NO quantification was performed using the Griess method, following the manufacturer’s instructions. Fifty microlitres of supernatant were collected from each culture, and nitrite (NO_2_^−^) production was measured. Nitrite is a stable metabolite derived from NO. Then, 50 μL of sulfanilamide solution was added and the mixture was incubated for 10 min, protected from light, at room temperature. A solution of 50 µL of NED (N-1-naphthylenediamine dihydrochloride) was added and the sample was incubated under the same conditions. Absorbance was measured using a spectrophotometer (Biotek EPOCH2) at 520 nm. The nitrite concentration was determined by interpolating the obtained values in the standard nitrite curve. Additional assays were performed to quantify the production of ROS and NO using the IC_50_ values of FuLt, DOX, PTX and 5-FU (Figures S1–S3).

### 4.10. Statistical Analysis

The data were analysed using GraphPad Prism 8.01 (San Diego, CA, USA). All results are representative of at least three independent experiments yielding similar results. Results are expressed as the mean ± SD. Statistical significance was assessed using a one-way for cytotoxicity assays and two-way ANOVA for oxidative assays. Differences were considered statistically significant when *p* < 0.05, represented as follows: *** *p* < 0.001, ** *p* < 0.01, * *p* < 0.05.

## 5. Conclusions

The present study demonstrated that FuLt acts synergistically with the chemotherapeutic agents DOX, PTX and 5-FU, significantly enhancing their anticancer activity and inhibiting the growth of homotypic spheroids. These combinations exhibited remarkable cytotoxic efficacy, inducing redox dynamics characterised by elevated ROS and NO production, particularly in heterotypic spheroids. In these models, immune cells present in splenocytes, such as macrophages, neutrophils, dendritic cells and lymphocytes, contribute to the formation of a tumour microenvironment that favours the activation of the immune response. The generation of ROS and NO supports the hypothesis that FuLt exerts an immunomodulatory effect in combination with the evaluated chemotherapeutic agents, with the potential to induce events compatible with immunogenic cell death. Together, these findings emphasise the therapeutic potential of FuLt and highlight the importance of exploring compounds derived from marine resources, such as *L. trabeculata*, for developing new cancer treatment strategies.

## Figures and Tables

**Table 1:** IC_50_ of *L. trabeculata* fucoidan and three chemotherapy drugs on TNBC 4T1 spheroids.

	IC_50_ (μg/mL)			
Time (h)	FuLt	DOX	PTX	5-FU
24	3	12	0.02	5
48	15	5	0.05	2
72	100	2	0.5	0.5

FuLt: fucoidan from *L. trabeculata*, DOX: doxorubicin, PTX: paclitaxel, 5-FU: 5-fluorouracil, and IC_50_: half-maximal inhibitory concentration. Note: The IC_50_ values for FuLt and PTX should be interpreted with caution, as their dose–response curves show an inverse proportionality to the applied concentration. This non-monotonic behaviour can produce apparent deviations in the IC_50_ values that do not reflect the progressive inhibition observed at later stages (Figure 1 and Figure 2).

**Table 2:** Concentration-response curves of the inhibitory effect of FuLt, DOX, PTX and 5-FU. Assays were performed on 4T1 spheroids following treatment for 24, 48 or 72 h.

Treatments							Parameters								
**FuLt.**	DOX	PTX	5-FU	*fa*			*m*	*Dm*	r	*m*	*Dm*	r	*m*	*Dm*	r
				24 h	48 h	72 h	24 h			48 h			72 h		
1				0.61	0.65	0.79	0.28	2.73	0.95	0.24	13.5	0.97	0.34	85.69	0.98
10				0.39	0.49	0.72									
100				0.21	0.44	0.52									
1000				0.2	0.24	0.27									
	0.01			0.02	0.11	0.03	0.53	11.4	0.99	0.36	6.5	0.91	0.56	2.16	0.95
	0.1			0.1	0.18	0.22									
	1.0			0.19	0.19	0.55									
	5.0			0.43	0.49	0.56									
	10.0			0.45	0.65	0.62									
		0.01		0.53	0.66	0.63	0.25	0.02	0.96	0.35	0.06	0.98	0.17	0.38	0.96
		0.1		0.36	0.39	0.61									
		0.5		0.36	0.38	0.47									
		1.0		0.32	0.27	0.44									
		5.0		0.19	0.2	0.38									
		10.0		0.15	0.12	0.38									
			0.01	0.33	0.06	0.38	0.07	10.8	0.99	0.4	4.73	0.93	0.22	0.17	0.95
			0.1	0.37	0.15	0.4									
			0.5	0.41	0.43	0.56									
			1.0	0.42	0.44	0.64									
			5.0	0.44	0.47	0.65									
			10.0	0.45	0.47	0.72									

FuLt: fucoidan from *L. trabeculata*, DOX: doxorubicin, PTX: paclitaxel, 5-FU: 5-fluorouracil, *fa*: fraction inhibition, *m*: slope of the median effect plot, *Dm*: median inhibitory concentration, r: linear correlation coefficient.

**Table 3:** Data summary of concentration-effect curves of binary combinations between FuLt and DOX against TNBC 4T1 spheroids, after 24, 48 and 72 h of treatment.

FuLt + DOX (μg/mL)					Parameters			DRI		Interaction
Hours	Ratio	Total Concentration	*fa*	CI Value	*m*	*Dm*	r	FuLt	DOX	
24	1:1	2	0.24	0.78				1.76 × 10^2^	1.29	**synergism ^1^**
		10	0.26	3.21	0.12	3.26 × 10^4^	0.92	2.39 × 10^1^	0.32	antagonism
		20	0.3	4.53				5.83	0.23	antagonism
	10:1	2	0.16	0.37				5.99 × 10^2^	2.73	**synergism ^2^**
		10	0.2	1.12	0.32	4.99 × 10^2^	0.98	4.49 × 10^1^	0.91	additive
		100	0.39	8.47				0.15	0.54	antagonism
	100:1	100	0.33	3.14				0.36	3.02	antagonism
		500	0.44	7.64 × 10^1^	0.35	8.16 × 10^2^	0.99	0.01	1.46	antagonism
		1000	0.53	5.59 × 10^2^				0.00	1.44	antagonism
	175:1	100	0.14	1.57				1.93 × 10^1^	0.66	antagonism
		175	0.14	5.58	0.10	4.80	0.66	5.44	0.19	antagonism
		500	0.17	5.52				1.69	0.20	antagonism
48	1:1	2	0.59	0.39				2.96	1.79 × 10^1^	**synergism ^3^**
		10	0.78	7.29 × 10^1^	0.62	1.15	1.00	0.01	4.32 × 10^1^	antagonism
		20	0.86	1.45 × 10^3^				0.00	9.86 × 10^1^	antagonism
	10:1	2	0.38	0.13				5.74 × 10^1^	9.28	**synergism ^4^**
		10	0.51	0.92	0.45	6.92	0.99	1.26	8.02	additive
		100	0.78	1.33 × 10^3^				0.00	23.74	antagonism
	100:1	100	0.48	5.44				0.19	5.29	antagonism
		500	0.73	2.33 × 10^3^	0.62	1.11 × 10^2^	1.00	0.00	2.06 × 10^1^	antagonism
		1000	0.79	1.85 × 10^4^				0.00	2.57 × 10^1^	antagonism
	175:1	100	0.21	3.37				3.43 × 10^1^	0.30	antagonism
		175	0.49	2.24 × 10^1^	1.08 × 10^5^	3.46 × 10^2^	1.00	0.05	2.94	antagonism
		500	0.61	2.38 × 10^2^				0.00	8.02	antagonism
72	1:1	2	0.45	0.67				1.55 × 10^2^	1.51	**synergism ^5^**
		10	0.76	2.03	0.90	2.56	1.00	0.58	3.42	antagonism
		20	0.87	3.16 × 10^1^				0.03	6.56	antagonism
	10:1	2	0.2	1.02				2.79 × 10^3^	0.98	additive
		10	0.42	0.79	0.75	1.37 × 10^1^	1.00	2.44 × 10^1^	1.33	**synergism ^6^**
		100	0.82	9.23 × 10^1^				0.01	3.62	antagonism
	100:1	100	0.45	1.30				1.56	1.52	antagonism
		500	0.72	9.35 × 10^1^	0.63	1.31 × 10^2^	0.99	0.01	2.38	antagonism
		1000	0.77	4.05 × 10^2^				0.00	1.91	antagonism
	175:1	100	0.2	3.15				5.10 × 10^1^	0.32	antagonism
		175	0.46	3.80	0.98	4.12 × 10^2^	1.00	0.39	0.81	antagonism
		500	0.55	1.14 × 10^1^				0.10	1.11	antagonism

FuLt: fucoidan from *L. trabeculata*, DOX: doxorubicin, *fa*: inhibitory effect, CI value: combinatorial index, *m*: slope of the median effect plot, *Dm*: median inhibitory concentration, r: linear correlation coefficient, DRI: Dose Reduction Index. CI = 1, additive effect; CI < 1, synergistic effect; and CI > 1, antagonistic effect. The superscript counts the synergistic indices in Table 3, Table 4, Table 5 and Tables S1–S3.

**Table 4:** Data summary of concentration-effect curves of binary combinations between FuLt and PTX against TNBC 4T1 spheroids, after 24, 48 and 72 h of treatment.

FuLt + PTX (μg/mL)					Parameters			DRI		Interaction
Hours	Ratio	Total Dose	*fa*	CI Value	*m*	*Dm*	r	FuLt	PTX	
24	2:1	2	0.16	0.05				8.29 × 10^2^	2.19 × 10^1^	**synergism ^7^**
		5	0.22	0.55	0.41	1.10 × 10^2^	1.00	8.11 × 10^1^	1.84	**synergism ^8^**
		10	0.27	3.25				1.53 × 10^1^	0.31	antagonism
	1:1	1	0.19	0.08				1.05 × 10^3^	1.28 × 10^1^	**synergism ^9^**
		2	0.2	0.20	0.21	1.34 × 10^3^	0.99	4.17 × 10^2^	4.96	**synergism ^10^**
		10	0.27	4.82				2.05 × 10^1^	0.21	antagonism
	10:1	2	0.19	0.03				2.88 × 10^2^	3.51 × 10^1^	**synergism ^11^**
		5	0.2	0.10	0.04	3.31 × 10^15^	0.90	9.17 × 10^1^	1.09 × 10^1^	**synergism ^12^**
		10	0.2	0.21				4.58 × 10^1^	5.46	**synergism ^13^**
	100:1	50	0.17	0.10				1.72 × 10^1^	2.21 × 10^1^	**synergism ^14^**
		70	0.26	1.10	0.85	2.94 × 10^2^	0.90	1.77	1.85	additive
		100	0.27	1.91				1.03	1.06	antagonism
48	2:1	2	0.21	0.25				2.56 × 10^3^	3.94	**synergism ^15^**
		5	0.23	0.89	0.17	4.99 × 10^3^	0.98	6.28 × 10^2^	1.13	**synergism ^16^**
		10	0.26	2.83				1.60 × 10^2^	0.35	antagonism
	1:1	1	0.29	0.65				1.13 × 10^3^	1.53	**synergism ^17^**
		2	0.29	1.31	0.02	2.70 × 10^17^	0.96	5.67 × 10^2^	0.77	antagonism
		10	0.3	7.51				9.28 × 10^1^	0.13	antagonism
	10:1	2	0.28	0.21				3.83 × 10^2^	4.84	**synergism ^18^**
		5	0.36	1.52	0.36	2.62 × 10^1^	1.00	3.28 × 10^1^	0.67	antagonism
		10	0.41	5.61				6.79	0.18	antagonism
	100:1	50	0.26	0.47				2.15 × 10^1^	2.38	**synergism ^19^**
		70	0.3	1.19	0.28	1.76 × 10^3^	0.86	6.70	0.96	antagonism
		100	0.3	1.70				4.69	0.67	antagonism
72	2:1	2	0.53	3.59				4.51 × 10^1^	0.28	antagonism
		5	0.55	1.44 × 10^1^	0.15	0.99	0.96	1.42 × 10^1^	0.07	antagonism
		10	0.59	7.53 × 10^1^				4.40	0.01	antagonism
	1:1	1	0.55	4.31				9.49 × 10^1^	0.23	antagonism
		2	0.56	1.09 × 10^1^	0.13	0.25	0.99	4.21 × 10^1^	0.09	antagonism
		10	0.62	2.36 × 10^2^				4.06	0.00	antagonism
	10:1	2	0.50	0.40				5.30 × 10^1^	2.64	**synergism ^20^**
		5	0.66	6.01 × 10^1^	0.65	2.02	0.99	2.68	0.02	antagonism
		10	0.73	8.43 × 10^2^				0.50	0.00	antagonism
	100:1	50	0.56	6.58				0.85	0.19	antagonism
		70	0.57	1.15 × 10^1^	0.30	2.39 × 10^1^	0.95	0.54	0.10	antagonism
		100	0.61	4.07 × 10^1^				0.23	0.03	antagonism

FuLt: fucoidan from *L. trabeculata*, PTX: paclitaxel, *fa*: inhibitory effect, CI value: combinatorial index, *m*: slope of the median effect plot, *Dm*: median inhibitory concentration, r: linear correlation coefficient, DRI: Dose Reduction Index. CI = 1, additive effect; CI < 1, synergistic effect; and CI > 1, antagonistic effect. The superscript counts the synergistic indices in Table 3, Table 4, Table 5 and Tables S1–S3.

**Table 5:** Data summary of concentration-effect curves of binary combinations between FuLt and 5-FU against TNBC 4T1 spheroids, after 24, 48 and 72 h of treatment.

FuLt + 5-FU (μg/mL)					Parameters			DRI		Interaction
Hours	Ratio	Total Dose	*fa*	CI Value	*m*	*Dm*	r	FuLt	5-FU	
24	2:1	2	0.31	2.82 × 10^2^				3.82 × 10^1^	0.00	antagonism
		5	0.35	6.24 × 10^1^	0.22	8.05 × 10^1^	1.00	7.98	0.02	antagonism
		10	0.39	1.29 × 10^1^				2.15 × 10^5^	0.08	antagonism
	1:1	1	0.3	3.99 × 10^2^				1.21 × 10^2^	0.00	antagonism
		2	0.41	1.32	0.19	3.88 × 10^1^	0.78	1.07 × 10^1^	0.82	antagonism
		10	0.42	4.06				1.84	0.28	antagonism
	10:1	2	0.19	4.69 × 10^5^				2.88 × 10^2^	0.00	antagonism
		5	0.35	1.72 × 10^1^	0.66	1.61 × 10^1^	0.97	5.85	0.06	antagonism
		10	0.4	2.67				1.36	0.52	antagonism
	100:1	50	0.33	6.25 × 10^1^				0.74	0.02	antagonism
		70	0.36	1.75 × 10^1^	0.62	1.64 × 10^2^	0.98	0.33	0.07	antagonism
		100	0.43	1.29 × 10^1^				0.08	2.48	antagonism
48	2:1	2	0.33	0.82				1.95 × 10^2^	1.23	**synergism ^21^**
		5	0.38	1.22	0.19	7.65 × 10^1^	0.99	3.13 × 10^1^	0.84	antagonism
		10	0.4	2.02				1.10 × 10^1^	0.52	antagonism
	1:1	1	0.31	0.77				7.62 × 10^2^	1.30	**synergism ^22^**
		2	0.4	0.59	0.26	1.61 × 10^1^	0.94	7.34 × 10^1^	1.73	**synergism ^23^**
		10	0.46	1.76				5.28	0.64	antagonism
	10:1	2	0.29	0.36				3.12 × 10^2^	2.83	**synergism ^24^**
		5	0.34	0.52	0.18	2.87 × 10^2^	0.96	4.74 × 10^1^	2.01	**synergism ^25^**
		10	0.35	0.94				1.97 × 10^1^	1.12	additive
	100:1	50	0.25	1.63				2.68 × 10^1^	0.63	antagonism
		70	0.3	1.35	0.50	4.34 × 10^2^	0.96	6.70	0.84	antagonism
		100	0.32	1.67				3.17	0.74	antagonism
72	2:1	2	0.56	1.30				3.16 × 10^1^	0.79	antagonism
		5	0.58	2.28	0.13	0.32	0.98	9.94	0.46	antagonism
		10	0.61	2.75				3.45	0.41	antagonism
	1:1	1	0.47	5.00				2.44 × 10^2^	0.20	antagonism
		2	0.57	1.61	0.24	1.10	0.92	3.74 × 10^1^	0.63	antagonism
		10	0.62	3.29				4.06	0.33	antagonism
	10:1	2	0.43	3.83				1.08 × 10^2^	0.26	antagonism
		5	0.49	3.19	0.25	6.07	1.00	2.12 × 10^1^	0.32	antagonism
		10	0.53	3.17				6.62	0.33	antagonism
	100:1	50	0.75	1.47 × 10^1^				0.07	5.43 × 10^1^	antagonism
		70	0.79	4.00 × 10^1^	0.41	3.25	0.94	0.03	1.10 × 10^2^	antagonism
		100	0.8	6.84 × 10^1^				0.01	1.02 × 10^2^	antagonism

FuLt: fucoidan from *L. trabeculata*, 5-FU: 5-fluorouracil, *fa*: inhibitory effect, CI value: combinatorial index, *m*: slope of the median effect plot, *Dm*: median inhibitory concentration, r: linear correlation coefficient, DRI: Dose Reduction Index. CI = 1, additive effect; CI < 1, synergistic effect; and CI > 1, antagonistic effect. The superscript counts the synergistic indices in Table 3, Table 4, Table 5 and Tables S1–S3.

## Data Availability

The authors declare that all relevant data supporting the findings of this study are available within the article and its Supplementary Materials files or from the corresponding authors upon request.

## Supplementary Materials

The following supporting information can be downloaded at: https://www.mdpi.com/article/10.3390/md23120451/s1, Table S1. Resume of CI values simulated by CompuSyn software, version 1.0, for binary combinations of FuLt and DOX. Table S2. Resume of CI values simulated by CompuSyn software, version 1.0, for binary combinations of FuLt and PTX. Table S3. Resume of CI values simulated by CompuSyn software, version 1.0, for binary combinations of FuLt and 5-FU. Figure S1. Relative ROS production. Figure S2. Nitrites production in 4T1 homotypic spheroids treated with IC_50_ FuLt: Fucoidan from *L. trabeculata* (100 µg/mL). Figure S3. Nitrites production in heterotypic spheroids of splenocytes and 4T1 cells treated with IC_50_ FuLt: Fucoidan from *L. trabeculata* (100 µg/mL), IC_50_ DOX: doxorubicin (2 µg/mL), IC_50_ PTX: paclitaxel (0.5 µg/mL), and IC_50_ 5-FU: 5-fluorouracil (0.5 µg/mL).
