# Supplementary material for: Synergistic Anticancer Activity of Fucoidan from Lessonia trabeculata Combined with Chemotherapeutic Agents in 4T1 Breast Spheroids
Source: Mar Drugs. 2025 Nov 26;23(12):451. doi: 10.3390/md23120451 (PMC12734982; doi:10.3390/md23120451)
Supplement: Supplementary file 1 [file marinedrugs-23-00451-s001.zip › marinedrugs-3877625-supplementary.pdf]

marine drugs

MDPI

Supplementary Materials

Synergistic Anticancer Activity of Fucoidan from *Lessonia trabeculata* Combined with Chemotherapeutic Agents in 4T1 Breast Spheroids

Rosa María Condori Macuri *, Libertad Alzamora-Gonzales *, Erasmo Honorio Colona-Vallejos, Raisa Teresa Cruz Riquelme, Laura Inés Pecho Chávez, Jherson Oscar Cisneros Gutierrez and Victor Alonso Montejo Anlas

Research Group Immunomodulators and Antitumor of Natural and Synthetic Origen, Immunology Laboratory, Universidad Nacional Mayor de San Marcos, Lima 11-0058, Peru; ecolonav@unmsm.edu.pe (E.H.C.-V.); raisa.cruz@unmsm.edu.pe (R.T.C.R.); laura.pecho@unmsm.edu.pe (L.I.P.C.); jherson.cisneros@unmsm.edu.pe (J.O.C.G.); victor.montejo1@unmsm.edu.pe (V.A.M.A.)

* Correspondence: rosa.condori@unmsm.edu.pe (R.M.C.M.); lalzamorag@unmsm.edu.pe (L.A.-G.); Tel.: +51-932675678 (R.M.C.M.); +51-986912733 (L.A.-G.)

Table S1. Resume of CI values simulated by CompuSyn software, version 1.0, for binary combinations of FuLt and DOX.

	Treatment (µg/mL)		CI			Interaction
**Ratio**	**FuLt**	**DOX**	**24 h**	**48 h**	**72 h**	
1:1	1.63×10^{4}	1.63×10^{4}	7.39×10^{3}			antagonism
	4.54×10^{5}	4.54×10	1.70×10^{2}			
10:1	8.08×10^{2}	8.08	2.96×10^{2}			antagonism
100:1	4.77×10^{9}	2.69×10^{7}	1.75×10^{9}			antagonism
175:1	0.57	0.57		0.13		**synergism**[26]
1:1	6.29	0.63		0.56		**synergism**[27]
10:1	1.09×10^{2}	1.09		8.27		**synergism**[28]
100:1	3.45×10^{2}	1.94		2.58×10^{1}		antagonism
175:1	1.28	1.28			0.61	antagonism
1:1	1.25×10^{1}	1.25			0.72	**synergism**[29]
10:1	1.30×10^{2}	1.30			2.11	**synergism**[30]
100:1	4.10×10^{2}	2.31			5.85	antagonism
175:0	1.63×10^{4}	1.63×10^{4}	7.39×10^{3}			antagonism
	4.54×10^{5}	4.54×10	1.70×10^{2}			

FuLt: fucoidan from *L. trabeculata*, DOX: doxorubicin, CI value: Combinatorial index. CI = 1, additive effect; CI < 1, synergistic effect; and CI > 1, antagonistic effect. CI with fa ≥ 0.5 are displayed. The superscript counts the synergistic indices in Tables 3, 4, 5, S1, S2, and S3.

Table S2. Resume of CI values simulated by CompuSyn software, version 1.0, for binary combinations of FuLt and PTX.

Treatment (μg/mL)			CI			Interaction
**Ratio**	**FuLt**	**PTX**	**24 h**	**48 h**	**72 h**	
2:1	7.30×10^{1}	3.65×10^{1}	1.84×10^{3}			antagonism
1:1	6.72×10^{2}	6.72×10^{2}	3.36×10^{4}			antagonism
10:1	3.01×10^{15}	3.01×10^{14}	1.60×10^{16}			**synergism**[31]
100:1	2.91×10^{2}	2.91	2.45×10^{2}			antagonism
2:1	3.33×10^{3}	1.66×10^{3}		2.87×10^{4}		antagonism
1:1	1.35×10^{17}	1.35×10^{17}		2.32×10^{18}		**synergism**[32]
10:1	2.38×10^{1}	2.38		4.24×10^{1}		antagonism
100:1	1.75×10^{3}	1.75×10^{1}		4.27×10^{2}		antagonism
2:1	0.66	0.33			0.88	**synergism**[33]
1:1	0.13	0.13			0.33	**synergism**[34]
10:1	1.84	0.18			0.51	**synergism**[35]
100:1	2.37×10^{1}	0.24			0.90	**synergism**[36]

FuLt: fucoidan from *L. trabeculata*, PTX: paclitaxel, fa: inhibitory effect, CI value: Combinatorial index. CI = 1, additive effect; CI < 1, synergistic effect; and CI > 1, antagonistic effect. CI with fa ≥ 0.5 are displayed. The superscript counts the synergistic indices in Tables 3, 4, 5, S1, S2, and S3.

Table S3. Resume of CI values simulated by CompuSyn software, version 1.0, for binary combinations of FuLt and 5-FU.

Treatment (μg/mL)			CI			Interaction
**Ratio**	**FuLt**	**5-FU**	**24 h**	**48 h**	**72 h**	
2:1	5.36×10^{1}	2.68×10^{1}	1.88×10^{1}			antagonism
1:1	1.94×10^{1}	1.94×10^{1}	6.90			antagonism
10:1	1.47×10^{1}	1.47	5.09			antagonism
100:1	1.63×10^{2}	1.63	5.63×10^{1}			antagonism
2:1	5.10×10^{1}	2.55×10^{1}		9.17		antagonism
1:1	8.05	8.05		2.30		antagonism
10:1	2.61×10^{2}	2.61×10^{1}		2.48×10^{1}		antagonism
100:1	4.30×10^{2}	4.30		3.27×10^{1}		antagonism
2:1	0.21	0.11			0.62	**synergism**[37]
1:1	0.55	0.55			3.18	antagonism
10:1	5.52	0.55			3.24	antagonism
100:1	3.21	0.03			0.22	**synergism**[38]

FuLt: fucoidan from *L. trabeculata*, 5-FU: 5-fluorouracil, fa: inhibitory effect, CI value: Combinatorial index. CI = 1, additive effect; CI < 1, synergistic effect; and CI > 1, antagonistic effect. CI with fa ≥ 0.5 are displayed. The superscript counts the synergistic indices in Tables 3, 4, 5, S1, S2, and S3.

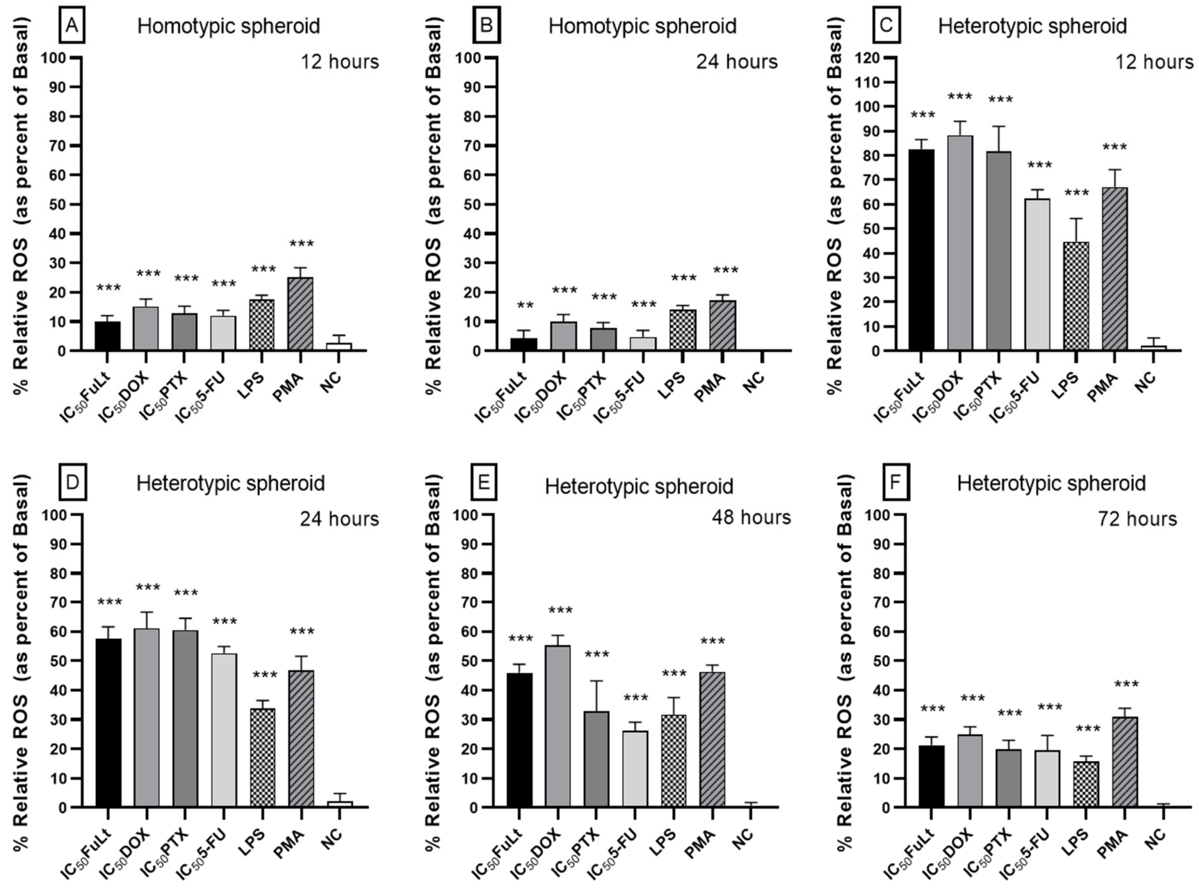

Figure S1. Relative ROS production. (**A**, **B**) In 4T1 homotypic spheroids. (**C**, **D**, **E**, **F**) In heterotypic spheroids of splenocytes and 4T1. All were treated with IC_{50} FuLt: Fucoidan from *L. trabeculata* (100 µg/mL), IC_{50} DOX: doxorubicin (2 µg/mL), IC_{50} PTX: paclitaxel (0.5 µg/mL), and IC_{50} 5-FU: 5-fluorouracil (0.5 µg/mL). LPS: lipopolysaccharides from *Escherichia coli* and PMA: phorbol myristate acetate were used as inducers of oxidative stress. NC: negative control (ROS production of untreated spheroids). Data are presented as mean ± SD (*n* = 6, the number of mouse spleens used is represented by *n*). *** p < 0.001; ** p < 0.01.

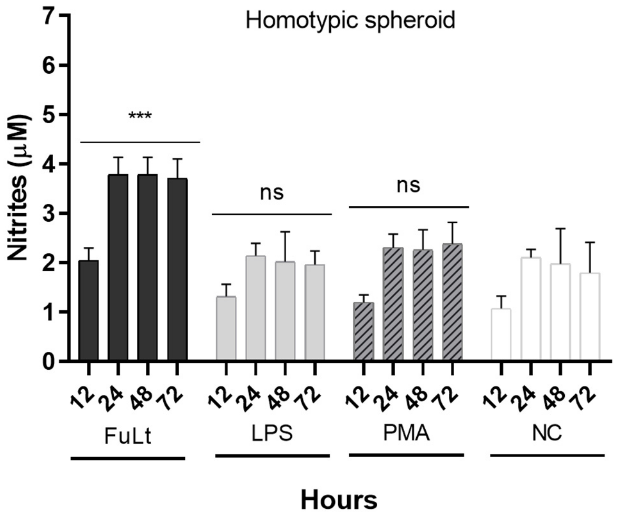

Figure S2. Nitrites production in 4T1 homotypic spheroids treated with IC_{50} FuLt: Fucoidan from *L. trabeculata* (100 µg/mL). LPS: lipopolysaccharides from *Escherichia coli* and PMA: phorbol myristate acetate were used as inducers of oxidative stress. NC: negative control (Nitrites production of untreated spheroids). Data are presented as mean ± SD (n = 6). *** p < 0.001.

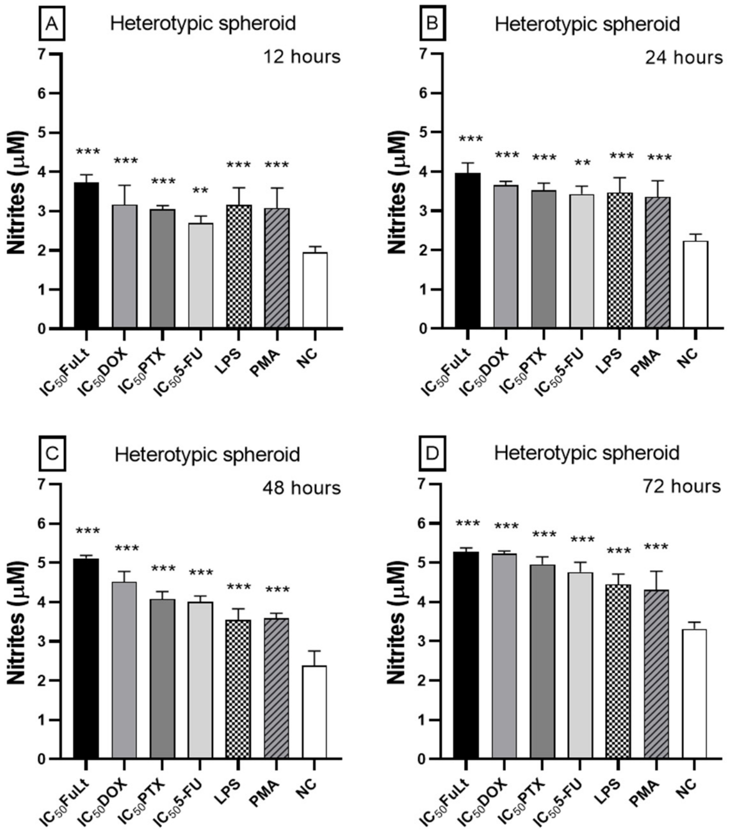

Figure S3. Nitrites production in heterotypic spheroids of splenocytes and 4T1 cells treated with IC_{50} FuLt: Fucoidan from *L. trabeculata* (100 µg/mL), IC_{50} DOX: doxorubicin (2 µg/mL), IC_{50} PTX: paclitaxel (0.5 µg/mL), and IC_{50} 5-FU: 5-fluorouracil (0.5 µg/mL). (**A**) 12 h, (**B**) 24 h, (**C**) 48 h and (**D**) 72 h. LPS: lipopolysaccharides from *Escherichia coli* and PMA: phorbol myristate acetate were used as inducers of oxidative stress. NC: negative control (Nitrites production of untreated spheroids). Data are presented as mean ± SD (n = 6). *** $p < 0.001$; ** $p < 0.01$.
